# Comparative Analysis of the Diagnostic Effectiveness of SATRO ECG in the Diagnosis of Ischemia Diagnosed in Myocardial Perfusion Scintigraphy Performed Using the SPECT Method

**DOI:** 10.3390/diagnostics12020297

**Published:** 2022-01-25

**Authors:** Łukasz Jerzy Janicki, Wiesław Leoński, Jerzy Stanisław Janicki, Mateusz Nowotarski, Mirosław Dziuk, Ryszard Piotrowicz

**Affiliations:** 1 Institute for Physico-Medical Research, Primax Medica S.A., 62-040 Puszczykowo, Poland; lukasz.janicki@satroecg.pl (Ł.J.J.); jerzy.janicki@satroecg.pl (J.S.J.); 2 Institute of Physics, University of Zielona Góra, 65-417 Zielona Góra, Poland; m.nowotarski@if.uz.zgora.pl; 3 Joint Laboratory of Optics of Palacký University and Institute of Physics of CAS, RCPTM, Faculty of Science, Palacký University, 77147 Olomouc, Czech Republic; 4 Department of Nuclear Medicine, Military Institute of Medicine, 04-141 Warsaw, Poland; mdziuk@wim.mil.pl; 5 Department of Cardiac Rehabilitation and Noninvasive Electrocardiology, National Institute of Cardiology Stefan Cardinal Wyszyński, State Research Institute, 04-628 Warsaw, Poland; ryszard4909@gmail.com

**Keywords:** ischemic heart disease, coronary heart disease, model of the electrical work of the heart, partial potentials, etiology of the QRS complex, electrocardiography, SPECT perfusion scintigraphy

## Abstract

There is a great need for early diagnosis of ischemic heart disease (IHD), the most common cause of which is haemodynamic disorders caused mainly by obstructive atherosclerosis of the coronary arteries. The diagnosis of IHD is usually made with the use of functional tests, which include resting ECG (R) or examination of significant perfusion disorders during exercise using the SPECT method. Despite the fact that the ECG (R) test is commonly used in cardiological diagnostics, it has a limited diagnostic value, especially in people with a low probability of coronary artery disease (CAD). In order to increase the effectiveness of the ECG (R) examination, SATRO ECG software, based on the single fibres heart activity model (SFHAM), was used to evaluate the electrocardiograms. The introduction of new classifiers from the available medical data to the analysis made it possible to evaluate the diagnostic efficacy of SATRO ECG (TOT) in predicting significant perfusion disorders in the exercise SPECT (TOT 2). These disorders are most often caused by obstructive atherosclerosis of the coronary arteries, which is the main cause of CAD. The database of 316 patients (219 men and 97 women, aged 57 ± 10 years) was analyzed using resting and stress ECG, perfusion scintigraphy performed using the SPECT method, and SATRO ECG analysis. The diagnostic efficacy parameters of SATRO ECG (TOT) in predicting significant perfusion abnormalities in the exercise-induced SPECT (TOT 2) study were: sensitivity, 99%; specificity, 91%; concordance, 96%; and positive, 96%, and negative, 97%, predictive values. The Kappa–Cohen coefficient was 0.92, and the statistical significance coefficient was *p* < 0.001. These results indicate a statistically significant agreement in the diagnosis of IHD in both diagnostic methods used.

## 1. Introduction

The method of imaging the variability of the electric potential generated by the heart is widely used in cardiological diagnostics as a functional test, e.g., to diagnose ischemic heart disease (IHD), the most common cause of which involves haemodynamic disorders caused mainly by obstructive coronary artery disease (CAD) [1,2]. However, the diagnostic efficiency of the ECG is unsatisfactory, because the current method of analyzing the measured potential does not allow one to recognize both the process of discrete stimulation of individual areas of the heart muscle (HM) and the potential occurring during the depolarization of individual parts of the effective HM fiber representing a specific area of HM [3]. In the current ECG studies, it is also difficult to determine the effect on the parameters of the potential of factors related to, for example, permanent loss of HM cell potential, disturbances in the intraventricular conduction system (ICS), or HM inflammation [4,5], which significantly reduces the effectiveness of IHD diagnosis using this tool. Most often, the ECG assessment takes into account the parameters of characteristic waves, segments, and their mutual relations. For example, the diagnosis of IHD often results from the assessment of the parameters of the potential associated with repolarization [6,7,8,9]. This test has limited diagnostic value, especially if the ECG was performed during the pain-free period in people with a low probability of developing CAD. This problem concerns tests performed both during rest ECG (R) and during exercise ECG (S) [10]. In recent years, the diagnostic possibilities of ECG have been expanded by using high-resolution analysis (HFQRS) [11,12,13,14] to determine the parameters of the QRS complex. Algorithms based on numerical transformations, machine learning, and other signal processing techniques have also been introduced. It is impossible to determine the etiology of heart disorders and their influence on the measured potential appearing on the chest surface during HM depolarization. ECG examination is common and easy to perform, therefore increasing its diagnostic effectiveness in the diagnosis of IHD is extremely important for general practitioners and cardiologists.

The emerging problem of the efficiency of ECG (R) was the motivation to develop a new model of the electrical activity of the heart, using the idea of the effective working fibers SFHAM described in [3]. The practical implementation of this model was carried out using SATRO ECG software, which is a medical device [15], and the algorithms used in it enable the automatic calculation of the parameters of each of the five partial potentials that appear during the depolarization of left ventricular HM cells: the interventricular septum (IS) and the anterior (AW), inferior (IW), lateral (LW), and posterior (PW) walls [3,16]. The method of analyzing the resultant ECG (R) potentials with the use of SFHAM has already been described in detail in the patent application [17].

In order to assess the effectiveness of SATRO ECG (TOT) in the diagnosis of IHD, mainly caused by obstructive CAD, new classifiers based on real medical data were introduced into the analysis, which the dynamics of the depolarization process in each of the above-mentioned parts of the HM. On the other hand, in the resting-exercise SPECT (TOT 2) examination, significant disturbances of HM perfusion were assessed in accordance with the current ESC guidelines [18], and the results of this method were adopted as the “gold standard” in the diagnosis of ischemic heart disease. Perfusion disorders caused by HM ischemia affect the course of the depolarization wave and the emerging electrical potentials. In line with this fact, it was hypothesized that the potential parameters calculated during the SATRO ECG (TOT) analysis should reflect these changes and enable the diagnosis of significant disturbances in the electrical activity of the heart.

Therefore, the aim of this study was to assess the diagnostic efficacy of SATRO ECG (TOT) in the detection of IHD diagnosed on the basis of the assessment of significant perfusion disorders during rest-exercise SPECT (TOT 2).

## 2. Material and Methods

The material was a medical database of 323 patients (197 men and 126 women) collected during preclinical studies to assess the diagnostic efficacy of SATRO ECG in predicting regional HM perfusion disorders diagnosed by resting and exercise SPECT. Study participants were referred by cardiologists after diagnosis of CAD or suspected CAD. At that time, there was no reliable information regarding possible changes in the heart function in the form of diagnosed HM perfusion disorders. Due to the SPECT exercise test, patients with absolute contraindications were excluded from the study, according to the guidelines [19,20], which include: the following: unstable angina with pain within the last 48 h, exacerbated heart failure, a history of myocardial infarction within the last 2–4 days, uncontrolled arterial hypertension above 220/110 mmHg, pulmonary hypertension, life-threatening arrhythmias, advanced atrioventricular blocks (without a pacemaker), acute pericarditis, or myocarditis. Patients who previously had percutaneous coronary intervention or had a history of coronary bypass surgery or a history of myocardial infarction were also included in the study. The information obtained can be used to evaluate short and long-term results in accordance with the guidelines [19,21]:

- revascularization (CABG or PCI);
- pharmacological therapy used to prevent ischemia or modify the profile of lipids and other factors influencing the development of atherosclerotic plaque;
- previously diagnosed pathological changes;
- lifestyle changes.

The patients were then referred to the Military Institute of Medicine in Warsaw (76 participants) or the Institute of Cardiology in Anin (247 participants). The research was conducted in accordance with the methodology approved by the relevant bioethical committees of the Military Medical Chamber in Warsaw, No. 54/2002, and the Bioethics Committee of the Institute of Cardiology in Warsaw, No. 740/2003.

Documentation in the database includes the characteristics of all patients and the results in digital form: standard ECG (R), SATRO ECG analysis, stress test ECG (S), and perfusion scintigraphy performed using the SPECT method at rest and exercise. Only SATRO ECG results were automatically calculated and interpreted as positive or negative and submitted directly to the study coordinator.

Inclusion criteria:

- age (30–80) years;
- sinus rhythm in the ECG recording;
- possession of a complete medical documentation containing at least the results along with the interpretation of ECG (R) tests, SATRO ECG analyzes, EKG (S), and rest and exercise perfusion scintigraphy performed using the SPECT method.

Exclusion criteria (meeting one of the conditions excluded the patient from the study):

- test transformed into pharmacological stress—only in the ECG (S) test;
- left bundle branch block (LBBB);
- disturbances of the intraventricular conduction system;
- hypertrophic or dilated cardiomyopathy;
- lack of informed consent of the patient to participate in the study;
- presence of a cardioverter or cardiac defibrillator.

The above-mentioned contraindications for exercise SPECT, which excluded patients from the study, and the inclusion and exclusion criteria, were selected in such a way that the SATRO ECG analysis (in particular, the QRS complex) was not influenced by other non-coronary factors to which they belong, including inflammation or permanent loss of the potential of HM working cells. There was also no information in the available database regarding patients who could possibly have unstable angina, most often diagnosed during a medical interview.

**Figure 1:**
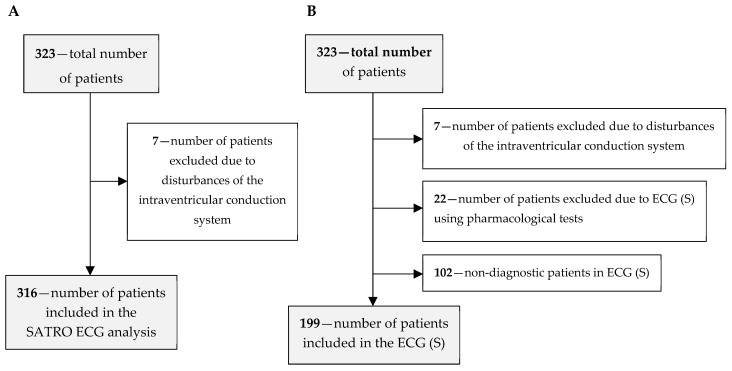
Flow of study participants for the assessment of diagnostic effectiveness: (**A**) without the results of the ECG (S) test, (**B**) for all diagnostic methods.

The diagnostic efficiency of SATRO ECG compared to resting-exercise SPECT was determined on a group of 316 patients (219 men and 97 women); the patient flow is shown in Figure 1A. Seven patients with conduction disturbances that had an influence on the parameters of the resultant potential measured on the thoracic surface were excluded; these were analyzed and described in the paper [22]. Changes in the parameters of the QRS complex are also a consequence of the ICS influence on the parameters of partial potentials during the depolarization of individual HM regions.

As part of the study, the diagnostic efficiency of the ECG (S) was also assessed on a group of 199 patients from the base; the patient flow is shown in Figure 1B. In this case, 22 patients, in whom the ECG (S) was performed after the use of pharmacological agents, were additionally excluded along with 102 patients, in whom the result of the ECG (S) test was non-diagnostic (in accordance with the accepted principles of evaluation of the test results). The above-mentioned exclusions were made due to the low diagnostic effectiveness of the ECG (S) examination in the case of using pharmacological agents [15].

The evaluation of the results of the SPECT exercise test (TOT2) did not exclude the results of pharmacological agents that do not adversely affect the diagnosis of CAD. On the other hand, studies conducted so far [23] show their beneficial influence on the effectiveness of CAD diagnosis in relation to invasive angiocardiography. The sensitivity and specificity of the diagnosis of CAD in the SPECT study after exercise is 73–92% and 63–87%, respectively, while in SPECT after pharmacological stress it is 90–91% and 75–84%, respectively, for strictures (>50%) of the coronary arteries. These data come from the 2013 ESC guidelines for the management of stable CAD [24]. Table 1 presents information from the medical database describing the patients whose results were qualified for further analysis.

### 2.1. Perfusion SPECT Scintigraphy

For the purposes of this study, the presence of significant perfusion abnormalities in the SPECT rest and exercise test (TOT 2) was adopted as the “gold standard” in the diagnosis of ischemic heart disease. These results included evaluation of the left ventricular (LV) cross-section and evaluation of pooled perfusion images presented as BULL’S-EYE maps. Radioisotope perfusion studies were performed using SPECT tomography using a one-day protocol after Tc-99m MIBI application—at rest and after exercise. In the morning, a resting test was performed (Tc-99m MIBI—0.1 mCi/kg bw was administered intravenously, and the data was recorded after about 1 h), after 3–4 h. the test was performed (exercise on a treadmill, intravenous Tc-99m MIBI was administered at the peak moment of exercise—0.3 mCi/kg bw, and then data recording after approx. 1 h). The tests were carried out with the use of a 2-head AXIS gamma camera (Picker/Philips), coupled with an Odyssey computer system. The second center (Military Institute of Medicine) used a dual-head Varicam camera coupled with an X-pert Pro data processing station and a Hermes system (Nuclear Diagnostic). SPECT acquisition conditions: matrix, 64 × 64; rotation angle, 180 degrees; number of projections, 68 collimator—high resolution, low energy, and parallelogram. For the reconstruction of the SPECT studies, a filtered back-projection algorithm, a Butterworth pre-reconstruction filter with a cutoff frequency of 0.4 of the fifth-order Nyquist frequency, was used. Correction of field of view heterogeneity and the center of rotation of the gamma camera have been improved. Correction of diffusion and suppression of radiation in the patient’s body was not applied. Myocardial Perfusion Imaging (MPI) was performed with a Tc-99m-MIBI radiotracer using single photon emission computed tomography (SPECT), which is a diagnostic technique with an established position among non-invasive imaging methods [18].

The well-documented clinical indications for the use of exercise stress test (SSS) in SPECT include assessment of cardiac function, mainly due to obstructive CAD and cardiovascular risk. Moreover, the SSS is of significant prognostic importance: the correct result of the exercise perfusion test (very low SSS value) defines low-risk patients (death or infarction occurs in <1% of patients annually in several years of follow-up); the greater the stress test perfusion impairment, the greater the risk of cardiovascular events. To compare the effectiveness of SATRO ECG in the diagnosis of significant HM perfusion disorders, the SPECT (TOT2) study used 4 anatomical areas (interventricular septum and anterior, interior, lateral walls), each of which consisted of 4 segments [3,16].

A positive global SPECT score (TOT 2) was defined as the presence of a significant exercise perfusion disorder (SSS ≥ 4) and the difference between the magnitude of rest and exercise perfusion disorders (SSS−SRS ≥ 1). Meeting these conditions determined HM ischemia, which was mainly due to the presence of stable CAD with obstructive atherosclerosis of the coronary arteries.

The area of impaired marker collection in exercise, revealed in SPECT studies, when properly stored in the resting place, corresponds entirely to the transient HM ischemia and is associated with a significant narrowing of the blood vessel supplying a given region of the muscle (and with completely preserved viability in the studied region). Local resting perfusion abnormalities are associated with critical vasoconstriction or the presence of dead myocytes in a given region; a partial improvement in accumulation relative to exercise testing (“partial transient loss”) indicates that at least some of the tissue vitality is preserved in the region; no improvement (“permanent loss”) increases the likelihood of necrosis in that area. The assessment of perfusion abnormalities is relative, and in balanced three-vessel disease or left coronary stenosis, the presence of CAD may be under-range or not detected.

### 2.2. SATRO ECG Analysis

**Figure 2:**
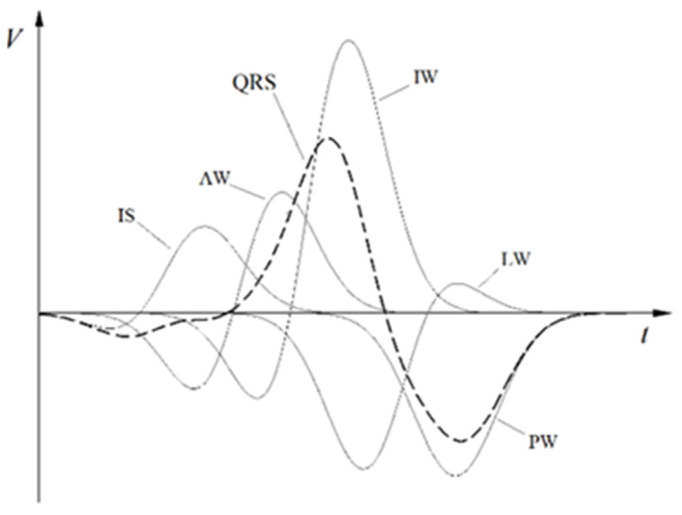
An example of the potential distribution for the Y coordinate (in the orthogonal Frank system related to the heart).

The results of the SATRO ECG (TOT) analysis contain information regarding the parameters of partial potentials appearing on the surface of the chest during the depolarization of individual areas of the myocardium, such as the interventricular septum (IS) and walls: anterior (AW), inferior (IW), lateral (LW), and posterior (PW). As an example, the partial potential distribution for the Y coordinate is shown in Figure 2.

The coordinates (X,Y,Z), calculated from the standard ECG signal (in particular from the QRS complex) are shown in Figure 2. The analysis of the resultant potentials (QRS complexes) for each lead is made on the basis of the averaged values, transformed into the Frank orthogonal system, the origin of which is located in the heart center, the X axis towards the patient’s left hand, the Y axis towards his feet, and the Z axis points on the patient’s back. According to the assumptions of the SFHAM model, the parameters of the independent partial potentials corresponding to five areas of the left ventricle were determined. Each of these areas is represented by one effective filament, along which we observe the flow of the electric charge wave. These flows lead to changes in the potential on the surface of the chest, which enables the calculation of the values of partial potentials described by the Formulas (1)–(3) and their duration determined by the Formulas (8)–(10).

The values of the normalized part potentials $\left(U_{1,i}\;,\;U_{2,i\;}\mathrm{and}\;U_{TOT}\right)\;$ were calculated on the basis of the relationships (1–3):

(1) $$U_{1,i}=\frac{A_{1,i}\;N_{TOT}}{N_{1,i}\;A_{TOT}}\times 100\%\;,$$

(2) $$U_{2,i}=\frac{A_{2,i}\;N_{TOT}}{N_{2,i}\;A_{TOT}}\times 100\%\;,$$

(3) $$U_{TOT}=\frac{A_{TOT}}{N_{TOT}}\times 100\%\;,$$

where $\left(N_{1,i},\;\;N_{2,i}\;\mathrm{and}\;N_{TOT}\right)$ are where the parameters determined for part potentials with normal electrical activity treated as the norm.

In the formulas the values $(A_{1,i}\;,\;A_{2,i\;}\mathrm{and}\;A_{TOT}$) were calculated according to the Formulas (4)–(6):

(4) $$A_{1,i}=\sum_{j=\left\{x,\;y,\;z\right\}}\int_{t_{0\left(1\right),i}}^{t_{k\left(1\right),i}}\left|V_{1,\;i,\;j}\right|dt\;,$$

(5) $$A_{2,i}=\sum_{j=\left\{x,\;y,\;z\right\}}\int_{t_{0\left(2\right),i}}^{t_{k\left(2\right),i}}\left|V_{2,\;i,\;j}\right|dt\;,\;$$

(6) $$A_{TOT}=\sum_{j=\left\{x,y,z\right\}}\int_{t_{0}}^{t_{end}}\left|V_{TOT}\right|dt,$$

where $\left(t_{0\left(1\right),i}\;\mathrm{and}\;t_{0\left(2\right),i}\right)$ and $\left(t_{k\left(1\right),i}\;\mathrm{and}\;t_{k\left(2\right),i}\right)$ denote the beginning and end of the appearing potentials with a cut-off point of $10^{-6}\;\left[V\right]$. Each of the part potentials is defined by two functions: $V_{1,i}\left(t\right)\;\mathrm{and}\;V_{2,i\;}\left(t\right),$ corresponding to the depolarization of a specific area of the left ventricular muscle, as shown in Figure 2., where $\left(N_{1,i},\;N_{2,i}\;\mathrm{and}\;N_{TOT}\right)$ are the parameters calculated for part potentials treated as the norm with normal electrical activity [3,16].

The duration of part potentials $\left(t_{1,i\;}\mathrm{and}\;t_{2,i}\right)$ was calculated according to the following formulas:

(7) $$t_{1,i}=\underset{j=\left\{x,\;y,\;z\right\}}{\sum }{\left({\left(t_{k\left(1\right)}-t_{0\left(1\right)}\right)}_{j}\right)}_{i}\;,$$

(8) $$t_{2,i}=\underset{j=\left\{x,\;y,\;z\right\}}{\sum }{\left({\left(t_{k\left(2\right)}-t_{0\left(2\right)}\right)}_{j}\right)}_{i}\;,$$

and the value of the normalized duration was calculated from the relationship:

(9) $$T_{Vp,i}\left[\%\right]=\;t_{Vp,i}\cdot 100\%/\;t_{Wp,i}$$

where:

(10) $$t_{Vp,i}=\left(t_{end,Vp,i}-t_{ο,Vp,i}\right)\;,$$

(11) $$t_{Wp,i}=\left(t_{end,Wp,i}-t_{ο,Wp,i}\right)\;,$$

determine the duration of each of the five potentials that appear during the depolarization of individual areas. In order to be able to assess the dynamics of depolarization, the J_i_ coefficients (12) were introduced, which is the ratio of the value of the potential occurring in the final depolarization phase (U_2, i_) to the initial value (U_1, i_) in each of the analyzed areas, where i = (IS, AW, IW, LW, PW) [3,16].

(12) $$J_{i}=\frac{U_{2,i}}{U_{1,i}}$$

The J_i_-factors were calculated from the data contained in the database for each patient. The values of the normalized part potentials $\left(U_{1,i}\;,\;U_{2,i\;}\mathrm{and}\;U_{TOT}\right)\;$ were calculated on the basis of the relationship (1–3).

SATRO ECG (TOT) analysis was interpreted as positive in the assessment of ischemia due to stable CAD with obstructive coronary atherosclerosis if the parameters of any of the four partial potentials appearing on the chest surface met the following conditions:

potent duration $\left(T_{V1,i}\;\mathrm{or}\;T_{V2,i}\right)>80\%$ and J−faktor<0.84 for any area of HM or resulting $U_{TOT\;}<45\%\;$ or part potentials $U_{1,i\;}or\;U_{2,i}$ were less than 15%.

### 2.3. Rest ECG

An ECG recorder by Medea from Gliwice PL (44–105) was used to measure the ECG, enabling the measurement of the electric potential on the chest surface from 10 leads with a sampling frequency of 500 Hz and analog filtration in the range of 0.03–100 Hz. The diagnosis of a positive ECG (R) result was made on the basis of the criteria specified in the AHA/ESC/WHF/NHLBI 2010 guidelines and also published in the American College of Cardiology (2018). There is information in the database where, for the recognition of a positive standard ECG result, a sharp criterion was used, assuming horizontal or downward oblique ST segment depression in at least one of the leads: I, II, aVL, aFV, V1–V6 (classes 4.2 and 4.3 Minnesota code).

The ECG (R) records were interpreted by an expert cardiologist who had no access to the rest of the data.

### 2.4. Stress ECG

The ECG exercise test was performed on a treadmill simultaneously with the radioisotope exercise test. ECG (S) has long been a method of assessing CAD and remains the initial test for most patients who can properly exercise with an interpretable electrocardiogram. For asymptomatic patients, the ECG (S) test was performed on the basis of the appropriate criteria provided in [10,25].

The result of the exercise ECG test was interpreted as positive if the following occurred during the test: (1) horizontal or downward oblique ST segment depression ≥ 1 mm at *J* point, (2) upward oblique ST segment depression ≥ 2 mm at a distance of 60 ms from the *J* point, (3) ST segment elevation ≥ 1 mm at *J* (leads without abnormal Q waves or QS complexes). 

The comparative assessment did not take into account the negative results of the stress test if the heart rate during the load did not reach 85% of the maximum heart rate, calculated according to the formula: 220−age of the examined person in years (questionable result of the stress test). The results of the stress test performed in patients with left bundle branch block were also not taken into account.

### 2.5. Diagnostic Effectiveness

The assessment of the diagnostic effectiveness of SATRO ECG (TOT) in relation to all the methods used was made on the basis of: sensitivity (Se), specificity (Sp), positive predictive value, PV (+), and negative predictive value, PV (−), according to the Formulas (13)–(16).

(13) $$\mathrm{Se}\left[\%\right]=\frac{\mathrm{TP}}{\mathrm{TP}+\mathrm{FN}}\times 100$$

(14) $$\mathrm{Sp}\left[\%\right]=\frac{\mathrm{TN}}{\mathrm{TN}+\mathrm{FP}}\times 100$$

Appropriate positive and negative predictive values:

(15) $$\mathrm{PV}\left(+\right)\left[\%\right]=\frac{\mathrm{TP}}{\mathrm{TP}+\mathrm{FP}}\times 100$$

(16) $$\mathrm{PV}\left(-\right)\left[\%\right]=\frac{\mathrm{TN}}{\mathrm{TN}+\mathrm{FN}}\times 100.$$

These were calculated from the number of true positive (TP), negative (TN), false positive (FP), and negative (FN) results for the methods under consideration.

The correlation of the results of the SPECT and SATRO methods was made using the statistical Cohen’s Kappa test, improved by Landis and Koch [26], who proposed threshold values of Cohen’s coefficients (another, simplified set of such thresholds was proposed by Cicchetti et al. [27]. As shown in the first test, if the Kappa coefficient is in the range of 0.61–0.80, the agreement between the two datasets is considered good, and if the Kappa becomes greater than 0.81, this agreement is “almost perfect”. In contrast, according to the second test, “perfect agreement” corresponds to a Kappa value greater than 0.75.

The SATRO ECG analysis is based on the ECG (R) measurement and concerns patients with the same pathological changes, so it was not necessary to consider the effect of the disease on the final result. In order to obtain full knowledge regarding IHD diagnostics, the effectiveness of SATRO ECG (TOT) in relation to ECG (R) and ECG (S) was also assessed in the studied patients.

## 3. Results

Taking into account the adopted criteria, the results of 316 patients (219 men and 97 women) were included in the research group, the characteristics of which are presented in Table 1.

In accordance with the adopted criteria, the frequency of occurrence of a positive result and the results consistent with the compared methods were determined (Table 2).

- SATRO ECG (TOT)—positive: 233/316 (74%) of patients
- SPECT (TOT 2)—positive: 227/316 (72%) of patients
- ECG (R—positive: 176/316 (56%) of patients
- ECG (S)—positive: 88/199 (44%) of patients.

The number of true positives and negatives was calculated against the SATRO ECG for each method. Then, the diagnostic efficacy parameters were determined for the comparison of SATRO ECG (TOT) to SPECT (TOT2); sensitivity, 99%; SATRO ECG (TOT) to ECG (R), 58%; ECG (S), 48%. The remaining results are shown in Table 3.

Table 4 presents the results of the Cohen’s Kappa test and the values of statistical significance (*p*) coefficients for the correlation of the SATRO ECG (TOT) results with the results of other methods.

The results of both methods are mainly influenced by the activity of myocardial cells and other non-coronary factors. The only difference is that perfusion scintigraphy assesses perfusion abnormalities in HM cells, and SATRO ECG (TOT) assesses electrical potential abnormalities during depolarization. The relationship between these disorders was described in the publication [3,16].

## 4. Discussion

In the previous studies presented in [15], the effectiveness of the SATRO ECG method in the diagnosis of IHD was assessed in comparison to the results of the resting and exercise SPECT test, obtaining a sensitivity of 100% and a specificity of 70%, as well as a Kappa–Cohen coefficient of 0.817 and a statistical significance of *p* < 0.001. These studies were carried out on a group of 243 people (168 men, 75 women).

In this study, the interpretation of the results obtained with the SATRO ECG method was extended to include the assessment of the value of the new *J* coefficient, defined by formula (12). According to the adopted assumptions, the reduction of the value of the partial potential appearing during the final phase of depolarization with its normal duration indicates that the main cause of IHD are haemodynamic disorders caused by obstructive atherosclerosis of the coronary arteries. The diagnosis of these changes, taking into account the aforementioned criteria, in any of the examined areas of HM was treated as a positive result, and the examination was marked with the acronym SATRO ECG (TOT). The overall evaluation of the SPECT study was determined on the basis of significant changes in exercise-induced per-fusion and was designated by the acronym SPECT (TOT 2). A positive global SPECT score (TOT 2) was defined as the presence of a significant exercise perfusion disorder (SSS ≥ 4) and the difference between the magnitude of rest and exercise perfusion disorders (SSS-SRS ≥ 1). Meeting these conditions defined HM ischemia, which was mainly due to the presence of stable CAD due to obstructive coronary atherosclerosis.

This method of evaluating the results and considering the contraindications to the SPECT exercise test, as specified in the guidelines [19,21], enabled the assessment of IHD caused mainly by coronary factors, mainly obstructive atherosclerosis of the coronary arteries. It is understood that obstructive coronary atherosclerosis is mainly diagnosed by anatomical studies such as invasive CAG or non-invasive CTA. It should also be emphasized that not every obstructive coronary atherosclerosis causes HM ischemic changes and vice versa. Our goal was not to diagnose atherosclerotic lesions but to recognize ischemic HM lesions confirmed by the assessment of significant SPECT perfusion disorders (TOT 2).

Moreover, the SPECT test is not free from defects (low specificity), but when used in appropriate groups of patients it allows the improvement of prognosis of patients with changes in the coronary vessels. In addition, there are more and more recent studies on the use of SPECT in various subgroups, in patients with diabetes, obesity or in women. In each of these cases, the study has been shown to be highly effective in diagnosing, differentiating, and forecasting the course of coronary artery disease [19,20,21]. For the group of patients with perfusion disorders in the SPECT exercise test, factors such as diabetes mellitus and the extent and size of the blood supply disorders allow grouping the results with a higher risk of death and possible benefits of revascularization.

Based on the obtained results, the diagnostic efficacy of SATRO ECG (TOT) in the diagnosis of significant perfusion disorders using exercise SPECT (TOT 2) was assessed. The calculated sensitivity (99%) and specificity (91%), with a Kappa–Cohen coefficient of 0.92 and a *p* value of <0.001, were assessed. The high diagnostic efficiency of SATRO ECG in the diagnosis of HM ischemic lesions is an important result of this study, as it may enable the use of the SATRO ECG test in the assessment of HM ischemia, while increasing the usefulness of the standard ECG test in the diagnosis of IHD.

HM ischemia is influenced by many diseases that, depending on their etiology, can be divided into coronary and non-coronary diseases. The first group includes, among others, CAD, which is mainly caused by obstructive atherosclerosis in the coronary arteries [2]. According to the ESC guidelines [24], the use of methods of cardiac function assessment significantly increases the scope of information regarding a patient’s condition and helps in making the correct decision regarding further treatment. For the initial diagnosis of obstructive CAD, functional tests (including ECG or exercise SPECT) and anatomical non-invasive (CAG) or invasive (ICA) tests are used. However, anatomical tests do not allow the assessment of ischemic changes, and thus do not diagnose cardiac dysfunction. It should be remembered that obstructive coronary atherosclerosis diagnosed in ICA is only one of the factors influencing the assessment of heart function. Coronary angiography focuses on the study of narrowing in the arteries (mainly epicardial), while SATRO ECG is a functional method that allows the assessment of electrical activity during depolarization of five areas of HM. Therefore, the combination of the results of both tests is extremely important, e.g., when making a decision regarding revascularization, information on both anatomy and ischemia is necessary [24].

In addition, it is extremely important that the SPECT (TOT2) assessment method adopted in this study allows for the determination of perfusion disorders caused by haemodynamic changes in the coronary arteries, e.g., obstructive CAD. Therefore, the high efficiency of SATRO ECG (TOT) compared to SPECT (TOT2) may mean that, in the future, this method of analysis will be able to indirectly identify changes in heart function caused by, e.g., obstructive CAD.

Continuing further research is necessary and very interesting because the etiology of changes in the electric potential appearing on the surface of the chest is not yet fully understood. In contrast, the pathophysiology of ischemic heart disease is complex and multifaceted and cannot be ascribed solely to the nature of obstructive CAD. Atherosclerosis is just one of many factors in the pathophysiological process, which can also include inflammation, thrombosis, dilated cardiomyopathy and impaired angiogenesis. Each of these elements may have a different effect on the electrical potential that arises during HM depolarization. On the basis of the calculated parameters of electrical activity, it is possible to evaluate most of these elements during the SATRO ECG analysis.

During the SATRO ECG analysis, many parameters of the electrical activity of the heart muscle are obtained, which in the future will allow the diagnosis of further pathological changes in the heart, such as:

haemodynamic disturbances in the coronary arteries,

haemodynamic disorders of the heart muscle,

inflammation of the heart muscle

The changes in electrical activity parameters observed in SATRO ECG are influenced by significant perfusion disorders diagnosed in SPECT (TOT 2), which enable the assessment of ischemic changes in the left ventricular muscle. The value of the electric potential, in particular during depolarization, depends on the amount of oxygen supplied to the interior of the HM cells by perfusion. The emergence of potential and perfusion disorders is closely related and relates to the processes taking place in HM cells. The analysis of standard ECG measurements based on the SFHAM model increases the diagnostic effectiveness of the ECG (R) in detecting ischemic changes that are not possible to assess in the traditional way of interpreting the results of this test. The adopted method of selecting the research group and the global evaluation of the SPECT and SATRO ECG results potentially makes it possible to diagnose ischemic heart disease caused mainly by obstructive atherosclerosis of the coronary arteries. There is a potential for the use of SATRO ECG as a functional test to assess the likelihood of coronary artery disease and to determine further patient management in accordance with ECS guidelines.

Janicki, J.S.; Janicki, Ł.J. Method for determining electrical activity of cardiac muscle. International patent application under the PCT Patent. PCT/IB2020/050639

## Figures and Tables

**Table 1:** Characteristics of 316 patients described in the database.

Characteristics of Patients	
Research has been carried out	2 April 2003–9 November 2007
Age (years)	57 ± 10
Men	219
Women	97
Diabetes mellitus	29 (9%)
Hypertension	138 (44%)
Hyperlipidemia	122 (39%)
History of a typical angina	31 (33%) ^2^
History of atypical angina	27 (29%) ^2^
No pain or no characteristic pain	36 (38%) ^2^
History of beta-blocker treatment	149 (47%)
Previous percutaneous coronary intervention	58 (18%)
Previous coronary bypass surgery	35 (11%)
Trans myocardial laser revascularization	10 (3%)
History of coronary angiography (2001–2003)	140 (44%)
History of myocardial infarction	115 (36%) ^1^
History of heart transplantation	2 (1%)
Conduction disturbances	7 (2%)
Mitral regurgitation	21 (7%)
Stress test performed using pharmacological tests	22 (7%)

^1^ including after 2 heart attacks 21 (7%), ^2^ results for 94 patients.

**Table 2:** The frequency of consistent results of the compared methods.

Compared Studies	Number of Patients	Result (+) (Number of Patients)	Result (-) (Number of Patients)	Corresponding Results (%)
SATRO ECG (TOT)/SPECT (TOT 2)	316	225	81	97
SATRO ECG (TOT)/ECG (R)	316	137	44	57
SATRO ECG (TOT)/ECG (S)	199	73	45	59

**Table 3:** Figures describing the diagnostic efficacy of SATRO ECG (TOT) in relation to other methods.

	**SATRO ECG(TOT)/ECG(R) (%)**	**SATRO ECG (TOT)/** **ECG(S) [%]**	**SATRO ECG (TOT)/** **SPECT(TOT2) [%]**
Sensitivity	58	48	99
Specificity	53	72	91
Compliance of the results	57	55	96
Predictive value PV (+)	77	82	96
Predictive value PV (−)	31	36	97

**Table 4:** The results of the Kappa-Cohen test and the values of statistical significance coefficients (*p*) for the correlation of SATRO ECG (TOT) with other methods.

	*p*-Value	Kappa–Cohen	Compatibility
SATRO ECG (TOT)/SPECT (TOT 2)	<0.001	0.92	YES
SATRO ECG (TOT)/ECG (R)	<0.003	0.10	NO
SATRO ECG (TOT)/ECG(S)	<0.001	0.18	NO

## Data Availability

The research presented in this paper was retrospective and the data collected from the database was anonymized.
